# Cutaneous tuberculosis of the dorsal aspect of the hand

**DOI:** 10.11604/pamj.2025.52.32.48192

**Published:** 2025-09-22

**Authors:** Amit Toshniwal, Alushika Jain

**Affiliations:** 1 Department of Respiratory Medicine, Datta Meghe Institute of Higher Education and Research, Wardha, Maharashtra, India,; 2 Department of Radiodiagnosis, Datta Meghe Institute of Higher Education and Research, Wardha, Maharashtra, India

**Keywords:** Cutaneous tuberculosis, ulcer, *Mycobacterium tuberculosis*

## Image in medicine

A 24-year-old male residing in a rural area of central India presented to the outpatient department with complaints of swelling over the right dorsal aspect of the hand for 15 days and pus discharge for 2 days, which was purulent in nature. On examination, the patient had a pulse rate of 80 beats per minute and a blood pressure of 100/60 mmHg. The patient had no history of addiction. Upon sending culture samples of purulent secretions, mycobacterial growth was observed, confirming the diagnosis of cutaneous tuberculosis. Daily dressing of the infected site was performed, and antitubercular therapy was started. The patient was advised to undergo monthly follow-up and have the infected site regularly dressed.

**Figure 1:**
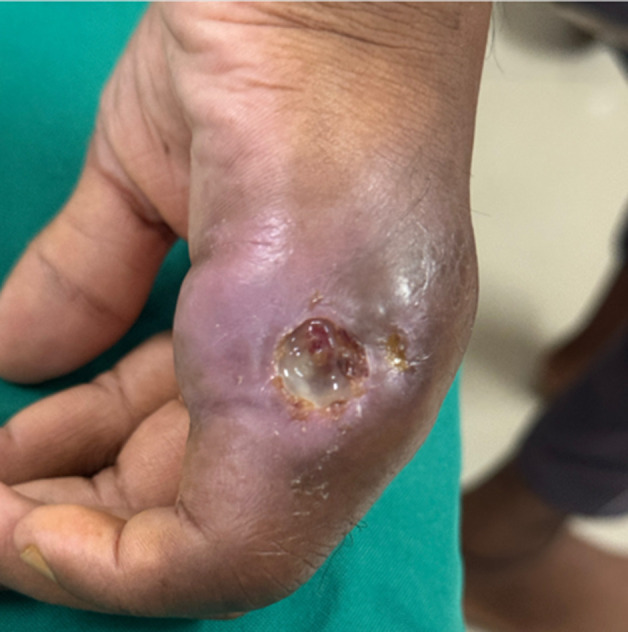
swelling over the dorsal aspect of the hand with purulent secretions

